# Development and validation of a diagnostic model for predicting cervical lymph node metastasis in laryngeal and hypopharyngeal carcinoma

**DOI:** 10.3389/fonc.2024.1330276

**Published:** 2024-04-26

**Authors:** Xingdong Wu, Yuhua Xie, Wanting Zeng, Xiaoping Wu, Jichuan Chen, Genping Li

**Affiliations:** ^1^ Department of Otolaryngology Head and Neck Surgery, Daping Hospital, Army Medical University, Chongqing, China; ^2^ Department of Critical Care Medicine, The Second Affiliated Hospital of Chongqing Medical University, Chongqing, China; ^3^ Department of Otolaryngology Head and Neck Surgery, The Second Affiliated Hospital of Chongqing Medical University, Chongqing, China

**Keywords:** laryngeal carcinoma, hypopharyngeal carcinoma, lymph node metastasis, diagnostic model, nomogram

## Abstract

**Objectives:**

The lymph node status is crucial for guiding the surgical approach for patients with laryngeal and hypopharyngeal carcinoma (LHC). Nonetheless, occult lymph node metastasis presents challenges to assessment and treatment planning. This study seeks to develop and validate a diagnostic model for evaluating cervical lymph node status in LHC patients.

**Materials and methods:**

This study retrospectively analyzed a total of 285 LHC patients who were treated at the Department of Otolaryngology Head and Neck Surgery, Daping Hospital, Army Medical University, from January 2015 to December 2020. Univariate and multivariate logistic regression analyses were employed to construct the predictive model. Discrimination and calibration were used to assess the predictive performance of the model. Decision curve analysis (DCA) was performed to evaluate the clinical utility of the model, and validation was conducted using 10-fold cross-validation, Leave-One-Out Cross Validation, and bootstrap methods.

**Results:**

This study identified significant predictors of lymph node metastasis in LHC. A diagnostic predictive model was developed and visualized using a nomogram. The model demonstrated excellent discrimination, with a C-index of 0.887 (95% CI: 0.835-0.933). DCA analysis indicated its practical applicability, and multiple validation methods confirmed its fitting and generalization ability.

**Conclusion:**

This study successfully established and validated a diagnostic predictive model for cervical lymph node metastasis in LHC. The visualized nomogram provides a convenient tool for personalized prediction of cervical lymph node status in patients, particularly in the context of occult cervical lymph node metastasis, offering valuable guidance for clinical treatment decisions.

## Introduction

Globally, head and neck squamous cell carcinoma is the seventh most frequent cancer. LHC have an annual incidence rate of 5.9 per 100,000 people in Europe, which translates to about 40,000 new cases per year (1).LHC are prone to cervical lymph node metastasis, particularly occult metastasis, significantly impacting patient prognosis, With 5-year relative survival rates of only 25% to 61% (1–3). Even in young patients, the 5-year overall survival rate can plummet to as low as 66%, with a recurrence rate of 62%, indicating a less than favorable prognosis (4). Studies (5, 6) have indicated that the overall lymph node metastasis rate among patients with LHC is as high as 59%, even among patients without clinically palpable cervical lymphadenopathy (CN0), there exists a notable risk of occult metastasis, estimated to be approximately 36% to 56%. Variations in metastatic patterns exist among patients with different tumor sites and stages. The remarkably high rate of lymph node metastasis and occult metastasis in LHC pose substantial challenges to clinical management, imposing significant economic and social burdens on patients, and profoundly affecting their prognosis (7).

Currently, the clinical assessment of cervical lymph nodes primarily encompasses imaging studies, ultrasound, and biopsy. The primary challenge encountered lies in accurately evaluating metastatic lymph nodes prior to treatment, particularly occult metastases. However, imaging may introduce concerns such as potential allergic reactions to contrast media and have limitations in the diagnosis of occult metastasis, the accuracy of fluorodeoxyglucose positron emission tomography–computed tomography(FDG-PET/CT) assessment is only 74%, and it carries risks such as radiation exposure and allergic reactions (8, 9). Furthermore, while ultrasound contributes to some extent to the evaluation of cervical lymph node status, its accuracy is relatively modest, even when combined with invasive biopsies, with an overall accuracy of only 77.4% (10). Preoperative biopsy also has limitations including low utilization rates, invasiveness, and the risk of biopsy failure (9). Accurate preoperative assessment of cervical lymph node status is critical, as incorrect assessments could impact subsequent patient care and treatment outcomes. As inadequate evaluation can lead to misguided treatment strategies for patients. Untreated metastatic lymph nodes can lead to shorter survival times and negative prognoses. Conversely, overtreatment such as performing neck dissection on patients without metastases may pose risks such as chyle leakage, recurrent laryngeal nerve injury, and even tracheostomy.

The current predictive models for assessing cervical lymph nodes are characterized by a singular approach, focusing solely on morphological features such as volume and thickness. They lack detailed consideration of important influencing factors such as age, tumor location, and T stage. Additionally, their clinical application is cumbersome, requiring assistance from ultrasound specialists in some cases, which presents inconvenience for clinicians. These limitations collectively constrain the practicality and accuracy of these models in clinical practice. Consequently, there is an urgent need for a more accurate and non-invasive approach to assess cervical lymph node status in patients with LHC. Such an approach would enhance diagnostic precision, facilitate tailored treatment strategies, and alleviate the physical and financial burdens on patients.

This study relies on clinical data from previous LHC patients within our department, aiming to construct a non-invasive predictive model based on patient clinical characteristics. The goal is to assist in a comprehensive assessment of cervical lymph nodes’ status, thereby providing valuable support for subsequent patient treatment decisions.

## Methods

### Data collection

As this is a retrospective study, sample size calculation was not conducted prior to data collection. We gathered all inpatient cases between January 2015 and December 2020 and then screened them based on inclusion and exclusion criteria. We aimed to include all eligible cases to enhance the reliability of statistical outcomes and analyses, as depicted in the flowchart. The variables chosen for analysis was based on clinical relevance and data availability, guided by clinical guidelines and input from oncology experts. This included commonly used clinical variables such as demographic characteristics (gender, age, smoking history, alcohol consumption), tumor features (tumor location, TNM staging), and pertinent hematological parameters like neutrophils and platelet counts, as advised by existing literature.

This study collected data from 322 patients at the Department of Otolaryngology Head and Neck Surgery, Daping Hospital, Army Medical University. Among them, 37 patients were excluded due to reasons such as treatment abandonment, readmission, or incomplete medical records. Therefore, the final analysis included 285 patients. The clinical data encompassed various patient characteristics and hematologic parameters. Hematologic parameters were assessed within one week before surgery. Based on these variables, we successfully established a clinical predictive model, which was subsequently evaluated and validated. Ultimately, we obtained a predictive model capable of non-invasively predicting cervical lymph node metastasis in patients with LHC. This study received approval from the Ethics Committee of the Army Medical Center of PLA [Approval Number: 2023 (170)] and adhered to the ethical principles outlined in the Declaration of Helsinki.

### Inclusion and exclusion criteria

Inclusion criteria: 1. Patients who were newly diagnosed and underwent surgical treatment; 2. Pathological diagnosis confirming malignancy. Exclusion criteria: 1. Patients with multiple systemic malignancies; 2. History of previous malignancies; 3. Incomplete medical records; 4. Presence of concurrent acute infections, hematologic disorders, or other conditions that could affect hematologic parameters. Patients were staged according to the 8th edition of the American Joint Committee on Cancer criteria.

### Statistical analysis

Data analysis was conducted using R version 4.2.2. Count variables were depicted as median (IQR), while categorical variables were represented as numerical (%). Employing the “glm” function, univariable logistic regression analysis was performed, with lymph node metastasis as the outcome variable and age, smoking index, neutrophil count, lymphocyte count, platelet count, NLR, PLR, hemoglobin, and albumin as predictor variables. Optimal cutoff values were identified using the maximum Youden index method (Youden index = sensitivity + specificity - 1). Subsequently, variables were transformed into categorical variables based on these cutoff values. Single-factor logistic regression analysis was then repeated using the transformed categorical variables along with other categorical variables as predictors. Variables with p < 0.05 were selected using backward stepwise multiple logistic regression to construct the final model. The “rms” function was utilized to create a nomogram visualizing the final model, which incorporated tumor location, T stage, neutrophil count, and platelet count.

**Figure 1:**
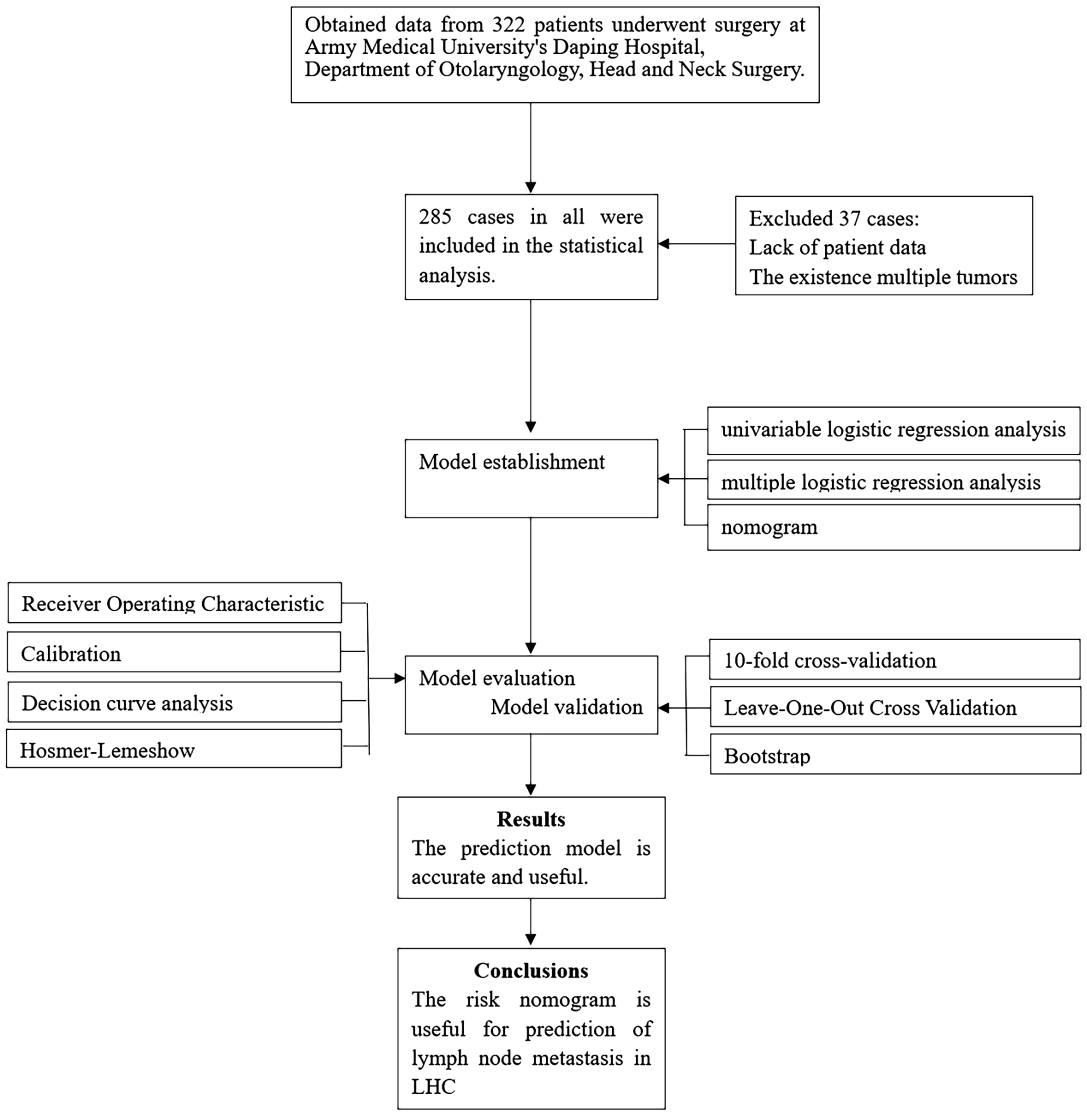
Flow diagram of study design.

Discrimination, calibration, clinical utility, and clinical impact of the model were assessed separately using receiver operating characteristic (ROC) curves generated by the “proc” package, calibration curves produced by the “rms” package, decision curves analysis, and clinical impact curves generated by the “rmda” package, respectively. Finally, the model underwent validation using 10-fold cross-validation, leave-one-out cross-validation, and bootstrap methods, please refer to the flowchart in **Figure 1** for details.

## Results

In the end, we included 285 patients for statistical analysis. Among them, the majority were male, accounting for 275 cases (96.5%), while there were only 10 female patients (3.5%), consistent with previous research findings (11). The median age of these patients was 61 years, with 245 cases (86.0%) having no lymph node metastasis and 40 cases (14.0%) having lymph node metastasis. Detailed clinical characteristics of the patients are presented in **Table 1**.

**Table 1:** Demographic and clinicopathologic characteristics of 285 patients.

Variables	Total (N=285)	Non-metastasis (N=245)	metastasis (N=40)
Age	61.00 (54.00,65.00)	61.00 (54.00, 65.00)	57.50 (52.75, 65.00)
Smoking history	600.00 (400.00,800.00)	600.00 (300.00, 800.00)	600.00 (400.00, 650.00)
Neutrophil	3.61 (2.96,4.30)	3.60 (2.88, 4.24)	3.67 (3.14, 5.17)
Platelet	185.00 (156.00,217.00)	182.00 (152.00, 215.00)	196.00 (176.00, 223.25)
Lymphocyte	1.82 (1.51,2.12)	1.82 (1.51, 2.12)	1.70 (1.33, 2.17)
NLR	2.00 (1.54,2.49)	1.97 (1.56, 2.46)	2.20 (1.52, 3.21)
PLR	101.27 (82.05,129.38)	99.99 (81.37, 127.19)	108.32 (89.85, 143.12)
Hemoglobin	145.00 (136.00,154.00)	145.00 (135.00, 154.00)	145.50 (136.00, 153.25)
Albumin	40.40 (38.00,43.00)	40.00 (38.00, 43.00)	41.45 (39.30, 43.55)
T stage			
T1	70 (24.6%)	70 (28.6%)	0 (0.0%)
T2	130 (45.6%)	122 (49.8%)	8 (20.0%)
T3	45 (15.8%)	36 (14.7%)	9 (22.5%)
T4	40 (14.0%)	17 (6.9%)	23 (57.5%)
Sex			
Female	10 (3.5%)	8 (3.3%)	2 (5.0%)
Male	275 (96.5%)	237 (96.7%)	38 (95.0%)
Tumor location			
Larynx	267 (93.7%)	240 (98.0%)	27 (67.5%)
Hypopharynx	18 (6.3%)	5 (2.0%)	13 (32.5%)
Smoking history			
No	41 (14.4%)	38 (15.5%)	3 (7.5%)
Yes	244 (85.6%)	207 (84.5%)	37 (92.5%)
Drinking history			
No	168 (58.9%)	146 (59.6%)	22 (55.0%)
Yes	117 (41.1%)	99 (40.4%)	18 (45.0%)
Hypertension			
No	241 (84.6%)	205 (83.7%)	36 (90.0%)
Yes	44 (15.4%)	40 (16.3%)	4 (10.0%)
Diabetes			
No	260 (91.2%)	222 (90.6%)	38 (95.0%)
Yes	25 (8.8%)	23 (9.4%)	2 (5.0%)
Pathology			
Non-SCC	281 (98.6%)	242 (98.8%)	39 (97.5%)
SCC	4 (1.4%)	3 (1.2%)	1 (2.5%)

Age, Smoking Index, Neutrophil, Platelet, Lymphocyte, NLR, PLR, Albumin, and Hemoglobin are presented as Median (IQR); Sex, T stage, Tumor location, Smoking history, Drinking history, Hypertension, Diabetes, and Pathology are presented as N (%). Percentages may not sum to 100% due to rounding. NLR, Neutrophil-to-Lymphocyte Ratio; PLR, Platelet-to-Lymphocyte Ratio; Smoking index: number of cigarettes smoked per day multiplied by the number of years of smoking.

We applied the maximum Youden index method to all numerical variables, found appropriate cutoff values, and transformed them into binary variables. See **Table 2** for details. These variables included age (≤60 vs. >60), smoking index (≤350 vs. >350), neutrophil count (≤5.0 vs. >5.0), platelet count (≤168 vs. >168), lymphocyte count (≤2.2 vs. >2.2), neutrophil-to-lymphocyte ratio (NLR) (≤2.5 vs. >2.5), platelet-to-lymphocyte ratio (PLR) (≤100 vs. >100), T stage (T1-2 vs. T3-4), albumin (≤40.5 vs. >40.5), and hemoglobin (≤150 vs. >150).

**Table 2:** Odds ratios (95% Confidence Intervals) and other statistical parameters obtained from logistic regression analysis using all patients.

Characteristics		univariate logistic regression analysis						multivariate logistic regression analysis					
		B	SE	OR	95%CI	Z	*P*	B	SE	OR	95%CI	Z	*P*
Age	(ref=“≤60”) >60	0.229	0.523	1.257	0.451-3.504	0.438	0.661						
Albumin	(ref=“≤40”) >40	0.666	0.351	1.947	0.979-3.874	1.899	0.058						
Diabetes	(ref=“No”) Yes	-0.677	0.758	0.508	0.115-2.244	-0.894	0.371						
Drinking history	(ref=“No”) Yes	0.188	0.343	1.207	0.616-2.363	0.547	0.584						
Hypertension	(ref=“No”) Yes	-0.563	0.555	0.569	0.192-1.69	-1.015	0.31						
Hemoglobin	(ref=“≤150”) >150	0.18	0.359	1.197	0.592-2.42	0.502	0.616						
Lymphocyte	(ref=“≤2.2”) >2.2	0.105	0.428	1.111	0.48-2.571	0.246	0.806						
Neutrophil	(ref=“≤5.0”) >5.0	1.804	0.417	6.072	2.681-13.75	4.325	<0.001	1.2	0.537	3.321	1.159-9.514	2.237	0.025
NLR	(ref=“≤2.5”) >2.5	0.858	0.358	2.358	1.169-4.756	2.398	0.016						
Pathology	(ref=“SCC”) Non-SCC	0.727	1.168	2.068	0.21-20.41	0.622	0.534						
Platelet	(ref=“≤168”) >168	1.295	0.462	3.651	1.476-9.03	2.805	0.005	1.132	0.546	3.102	1.064-9.046	2.074	0.038
PLR	(ref=“≤100”) >100	0.855	0.368	2.352	1.144-4.839	2.325	0.02						
Sex	(ref=“Female”) Male	-0.444	0.81	0.641	0.131-3.137	-0.549	0.583						
Tumor location	(ref=“Laryngeal”) Hypolarynx	3.14	0.564	23.111	7.651-69.808	5.568	<0.001	2.947	0.688	19.049	4.946-73.37	4.285	<0.001
Smoking history	(ref=“No”) Yes	0.817	0.626	2.264	0.664-7.722	1.306	0.192						
Smoking index	(ref=“≤350”) >350	0.906	0.5	2.475	0.929-6.595	1.814	0.07						
T stage	(ref=“T1-2”) T3-4	2.673	0.425	14.491	6.3-33.331	6.296	<0.001	2.686	0.496	14.673	5.55-38.791	5.412	<0.001

Ref, reference category; B, Coefficient; SE, Standard Error; OR, Odds Ratios; Z, standard score; NLR, Neutrophil-to-lymphocyte ratio; PLR, Platelet-to-lymphocyte ratio; Smoking index, Number of cigarettes smoked per day multiplied by the number of years of smoking.

In univariate logistic regression analysis, sex differences did not significantly impact lymph node metastasis (*p*=0.583, OR= 0.641, 95% CI: 0.131-3.137). Similarly, age, smoking history, smoking index, alcohol consumption history, lymphocyte count, hemoglobin, albumin, hypertension, diabetes, pathological type, and other factors did not show significant predictive value for lymph node metastasis in univariate analysis. However, we found that the following variables had predictive value for the presence of lymph node metastasis: tumor location (*p*<0.001, OR=23.111, 95% CI: 7.651-69.808), indicating that the probability of lymph node metastasis in hypopharyngeal cancer was approximately 23.1 times that of laryngeal cancer; T stage (*p*<0.001, OR=14.491, 95% CI: 6.300-33.331), indicating that the probability of lymph node metastasis in advanced-stage tumors (T3-4) was approximately 14.5 times that of early-stage tumors (T1-2); neutrophil count (*p*<0.001, OR=6.072, 95% CI: 2.681-13.75), indicating that the probability of lymph node metastasis was approximately 6.1 times higher in patients with high neutrophil counts (>5.0) compared to those with low neutrophil counts (≤5.0); platelet count (*p*=0.005, OR=3.651, 95% CI: 1.476-9.03), indicating that the probability of lymph node metastasis was approximately 3.6 times higher in patients with high platelet counts (>168) compared to those with low platelet counts (≤168); NLR (*p*=0.016, OR=2.358, 95% CI: 1.169-4.756), indicating that the probability of lymph node metastasis was approximately 2.4 times higher in patients with high NLR (>2.5) compared to those with low NLR (≤2.5); PLR (*p*=0.02, OR=2.352, 95% CI: 1.144-4.839), indicating that the probability of lymph node metastasis was approximately 2.4 times higher in patients with high PLR (>100) compared to those with low PLR (≤100).

The above univariate logistic regression analysis considered the impact of each variable on lymph node metastasis. To more accurately assess the influence of multiple variables on the outcome and their potential confounding effects, we employed a backward stepwise approach to introduce these variables into a multivariate logistic regression analysis to determine their contributions. In the final multivariate analysis, NLR and PLR were excluded, and the following variables were retained: tumor location (*p*<0.001, OR= 19.049, 95% CI: 4.946-73.37), T stage (*p*<0.001, OR=14.673, 95% CI: 5.55-38.791), neutrophil count (*p*<0.025, OR= 3.321, 95% CI: 1.159-9.514), and platelet count (*p*<0.038, OR= 3.102, 95% CI: 1.064-9.046). Ultimately, we established a cervical lymph node metastasis prediction model for LHC based on tumor location, T stage, neutrophil count, and platelet count as predictive factors. The Hosmer-Lemeshow test indicated a good model fit (*p*=0.352, Chi-square=3.266, df=3).

**Figure 2:**
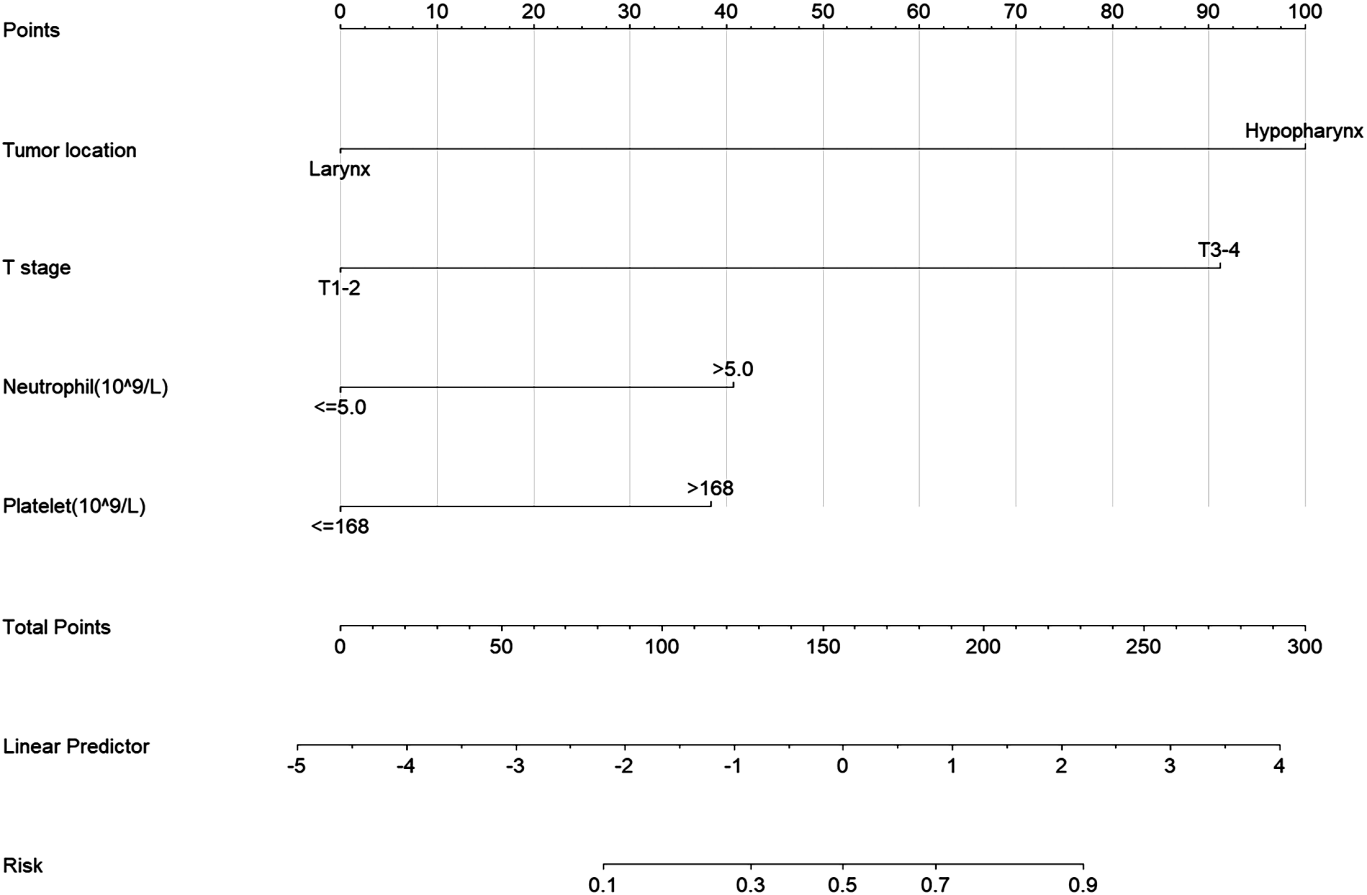
Nomogram for Predicting Neck Lymph Node Metastasis Risk in LHC. Tumor location, Primary tumor site; T stage, Primary tumor stage; Neutrophil, neutrophil count, Platelet, platelet count.

To personalize the prediction of cervical lymph node status in patients, we created a nomogram based on the model’s predictive factors, as shown in **Figure 2**. Using each patient’s Total points, we can predict their risk of cervical lymph node metastasis.

### Discrimination

**Figure 3:**
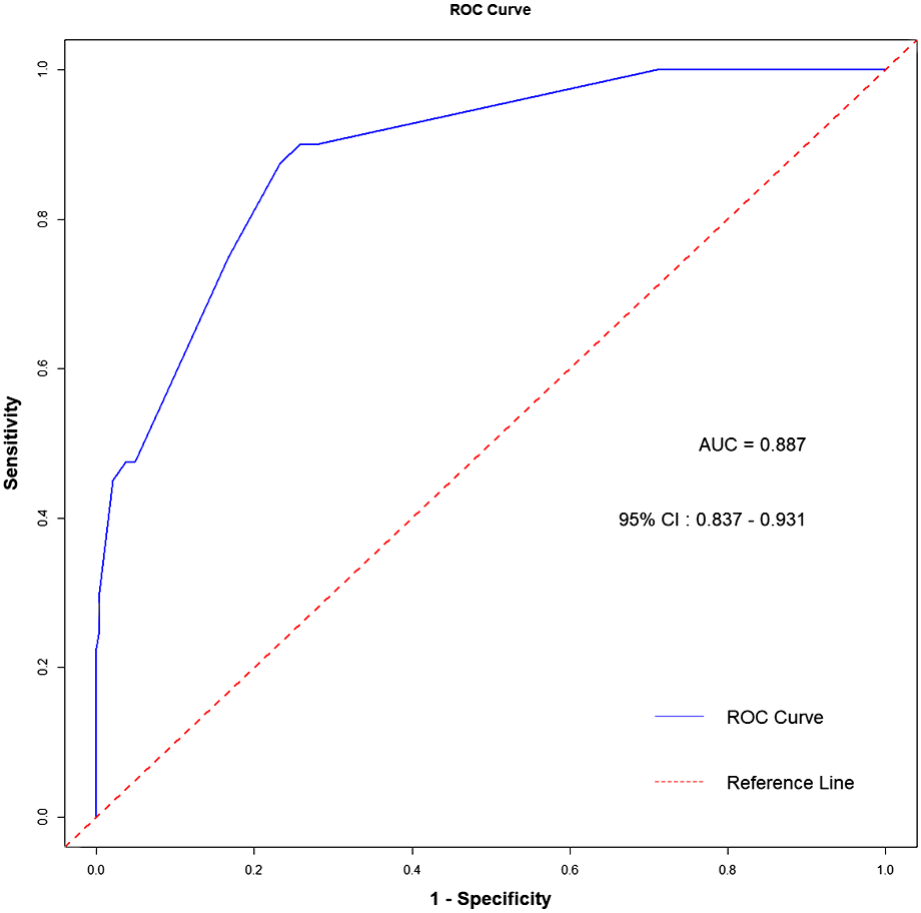
The ROC curve of the predictive model. AUC, Area Under the Curve.

We assessed the predictive model’s ability to distinguish between the lymph node metastasis group and the non-metastasis group using ROC curves. The results demonstrated that the prediction model constructed with tumor location, T stage, neutrophil count, and platelet count had excellent discrimination ability, with an Area Under the Curve (AUC) of 0.887, 95% CI: 0.835-0.933. The AUC value at this point is equivalent to the concordance index, as shown in **Figure 3**.

### Calibration

**Figure 4:**
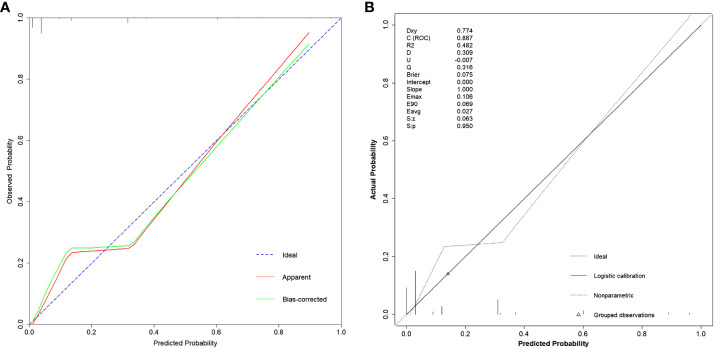
Calibration curves of the model under different functions. **(A)** Using the calibrate function, the bias-corrected line or the Apparent line approaching the Ideal line indicates higher consistency between predicted and actual values. **(B)** Using the val.prob function, Dxy represents the magnitude of correlation between predicted and actual values; Brier represents mean squared error, with smaller values indicating better calibration performance; *p*=0.950 indicating a good fit.

In **Figure 4A**, we utilized the Calibration curve to evaluate and visualize the disparity between the model’s predicted values and actual values. The horizontal axis represents predicted values, while the vertical axis represents actual values. The blue dashed line labeled “Ideal” serves as a reference line, the red solid line labeled “Apparent” represents the fit between the model’s predicted values and actual values, and the green solid line labeled “Bias-corrected” indicates the fit between the model’s predicted values and actual values after calibration. The shape of the curve suggests good consistency between the model’s predicted values and actual values. In **Figure 4B**, with Dxy=0.774, indicating a strong correlation between predicted values and actual values, and *p*=0.950, indicating good model fit.

### Clinical utility

**Figure 5:**
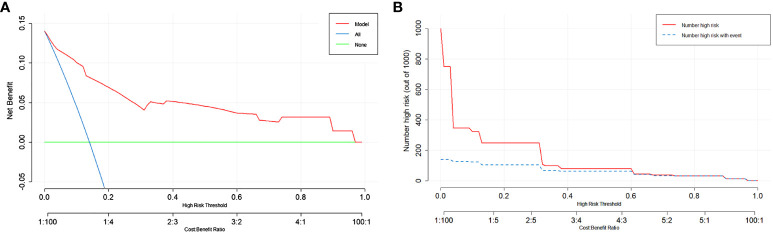
Evaluation of the predictive model using DCA and Clinical Impact Curve. **(A)** The y-axis represents the net benefit, and the x-axis represents the threshold probability. Within the range of 0-0.9, the DCA curve is located above the “None” and “All” lines, indicating the model’s suitability in this range. **(B)** The y-axis represents the number of individuals classified as high risk. The solid red line represents the number of individuals predicted as high risk by the model, while the dashed blue line represents the number of individuals predicted as high risk who experienced the outcome event. In the range where the x-axis exceeds 0.3, the two lines are close together, indicating the effectiveness of the model.

Next, we assessed the clinical utility of the model. We used DCA and Clinical Impact Curve to evaluate how patients benefit from the model. In **Figure 5A**, the x-axis represents the threshold probability values, and clinicians make selections based on the actual situation. The green horizontal line indicates that all patients have no metastasis and receive no intervention, resulting in a net benefit of 0. The blue diagonal line represents all patients having metastasis and receiving intervention, resulting in a linear net benefit. Both of these lines are considered ineffective. The red curve represents the DCA curve. Within the threshold probability range of 0 to 0.9, the DCA curve is positioned above both the None and All ineffective lines, indicating that the model adds clinical value to patients within these threshold probability values and demonstrates clinical utility. In **Figure 5B**, the Clinical Impact Curve demonstrates that when the horizontal axis exceeds 0.3, the solid line approaches the dashed line, indicating close alignment between model predictions and actual outcomes. Both figures suggest that the model has good clinical Utility.

**Figure 6:**
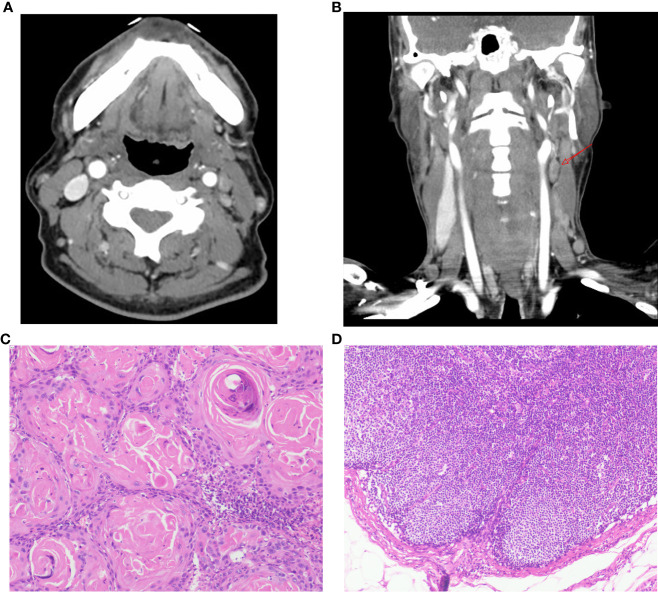
Patient’s Enhanced CT scans and Postoperative Pathology. **(A-B)** Axial and coronal CT images of the patient’s neck obtained after contrast enhancement. The images reveal an enlarged lymph node in the left level II area (arrow). The radiologist assessed this lymph node preoperatively, considering the possibility of lymph node metastasis associated with the tumor. **(C)** Pathological findings from postoperative tumor specimens show the presence of keratin pearls, indicative of squamous cell carcinoma (HE staining, 100x magnification). **(D)** Postoperative lymph node pathology results do not demonstrate the presence of cancer cells (HE staining, 100x magnification).

Our model has demonstrated remarkable efficacy in real-world clinical scenarios. Take, for example, a recent case involving a 62-year-old male patient diagnosed with laryngeal cancer (cT4N2M0) upon admission. Enhanced CT scans indicated the possibility of left cervical lymph node involvement. Consequently, the patient underwent a comprehensive procedure involving total laryngectomy and left neck lymph node dissection, with postoperative histopathology confirming the absence of lymph node metastasis (depicted in **Figure 6**). Notably, upon admission, the patient presented with a neutrophil count of 5.45 × 10^9/L and a platelet count of 159 × 10^9/L. According to our model’s calculations, the estimated risk of cervical lymph node metastasis for this patient was a mere 32.7% (95% CI: 0.118-0.638). This case underscores the strengths of our model in predicting neck lymph node status, thereby aiding clinical decision-making. The study indicates that current clinical assessment methods are no longer sufficient to accommodate the complex needs of patients (12, 13). In contrast, our model has the potential to prevent both over-treatment and under-treatment resulting from limited assessments.

### Model validation

This study employed three validation methods, including 10-fold cross-validation, Leave-One-Out Cross Validation, and Bootstrap, to ensure the robustness of the model. The results in **Table 3** show that all three methods indicate model accuracy of around 90%, with Kappa values exceeding 0.44 and the area under the ROC curve above 0.84. This confirms the model’s excellent generalization ability.

**Table 3:** Validation results of the model using three different methods: 10-fold cross-validation, leave-one-out cross validation, and bootstrap.

Method	Accuracy	Kappa	AUC	Sensitivity	Specificity
10-fold cross-validation	0.905	0.483	0.881	0.980	0.450
Leave-one-out cross-validation	0.905	0.522	0.840	0.980	0.450
Bootstrap	0.891	0.441	0.877	0.973	0.392

## Discussion

Our study successfully established and validated a diagnostic prediction model that can non-invasively predict neck lymph node status in patients before treatment, providing valuable information for their subsequent treatment. This model offers a more accurate and convenient method for lymph node assessment, avoiding the potential issues associated with traditional methods of lymph node evaluation, such as drug allergies, invasive procedures, suboptimal sample quality, and inadequate assessment (8, 12, 14).

Tobacco and alcohol are widely recognized as risk factors for head and neck tumors (15, 16), as they can damage mucosal cells, increase exposure to carcinogens, and promote tumor initiation and progression (17). However, the association of tobacco and alcohol with tumor cell metastasis to lymph nodes remains unclear (18–20). In univariate logistic regression analysis, our study results show no significant correlation between drinking history and neck lymph node metastasis (*p*=0.584, OR=1.207, 95% CI: 0.616-2.363), and smoking index (*p*=0.07, OR=2.475, 95% CI: 0.929-6.595) and smoking history (*p*=0.192, OR=2.264, 95% CI: 0.664-7.722) were also not confirmed as risk factors for lymph node metastasis.

Although we initially considered NLR and PLR as potential predictive factors, they were not included in the final prediction model in the multivariate logistic regression analyses. While neutrophils and platelets play important roles in tumor metastasis (21, 22), the roles of NLR and PLR in the field of cancer are still uncertain (23–25). While they showed a trend toward lymph node metastasis in univariate analysis, their impact was not as significant as tumor location, T stage, neutrophil count, and platelet count. Additionally, NLR and PLR are influenced by various factors, including medications and other disease states, which may introduce confounding variables (26). In contrast, tumor location, T stage, neutrophil count, and platelet count showed significant associations with neck lymph node metastasis in both univariate and multivariate logistic regression analyses. The multivariate results revealed that advanced-stage (T3-4) patients had a 15-fold higher risk of lymph node metastasis compared to early-stage (T1-2) patients (*p*<0.001, OR=14.673, 95% CI: 5.55-38.791), and the risk of neck metastasis for hypopharyngeal cancer was 19 times higher than that for laryngeal cancer (*p*<0.001, OR=19.049, 95% CI: 4.946-73.37). High neutrophil count (>5.0) was associated with a 3-fold higher risk of metastasis compared to low neutrophil count (≤5.0) (*p*=0.025, OR=3.321, 95% CI: 1.159-9.514), and high platelet count (>168) was associated with a 3-fold higher risk of metastasis compared to low platelet count (≤168) (*p*=0.038, OR=3.102, 95% CI: 1.064-9.046), as detailed in **Table 2**, indicating an association between these factors and neck lymph node metastasis.

Tumor staging is closely associated with lymph node metastasis, and as the T stage increases, the probability of lymph node metastasis also rises (27). In a study on occult lymph node metastasis in laryngeal cancer, the rate of occult lymph node metastasis was 15.4% for T2 supraglottic laryngeal cancer, while it was as high as 35.7% for T4 cases (28). Another study (29) pointed out that lymph node metastasis in hypopharyngeal cancer is closely related to tumor staging, with advanced cases (T3-T4) being more prone to lymph node metastasis than early cases (T1-2), which is consistent with our research findings. Research suggests that tumors in different locations exhibit significant differences in lymph node metastasis (11), particularly tumors in the hypopharynx, which are considered high-risk factors for lymph node metastasis, with a metastasis rate as high as 70% (30, 31). Additionally, hypopharyngeal cancer is more likely to metastasize to the tracheal lymph nodes (32). In contrast, the lymph node metastasis rate for laryngeal cancer is only 16.2%, even in T4-stage laryngeal cancer cases, the metastasis rate remains only 36% (33, 34), consistent with our predictive model, highlighting the significant impact of different tumor locations on the risk of lymph node metastasis. Neutrophils also play an important role in tumor metastasis (22, 35), as they can travel with circulating tumor cells and promote tumor cell proliferation by releasing cytokines, further validating this perspective in our study. Platelets can also participate in the tumor metastasis process in multiple ways, including promoting the survival of tumor cells, assisting in evading immune surveillance, and facilitating tumor cell adhesion to endothelial cells for penetration into capillaries for distant metastasis (36). Currently, drugs that intervene in the interaction between platelets and tumor cells are being explored to inhibit tumor metastasis (21), and our study results support this perspective. Unfortunately, the aforementioned studies primarily focused on analyzing individual factors in lymph node metastasis, without considering the synergistic effects of multiple factors in lymph node metastasis. This is precisely the highlight of our research.

We comprehensively employed various methods including discriminative analysis, calibration curve, Hosmer-Lemeshow test, and clinical utility to assess the performance of our model. The results demonstrated that our model excelled in all these metrics, displaying remarkable discriminative power, goodness of fit, and significant potential for clinical application. This underscores the importance of our predictive model in assessing neck lymph node status among LHC patients. Another highlight of our study is the multi-level validation conducted on the model, including 10-fold cross-validation, leave-one-out cross-validation, and bootstrap methods. This not only helps prevent model overfitting but also provides a comprehensive evaluation of its generalization ability. Consistent validation results across different methods consistently indicated outstanding accuracy and stability, affirming the model’s strong generalization potential.

Currently, there is limited research on predictive models specifically targeting occult lymph node metastasis in laryngeal and hypopharyngeal carcinoma. Previous models have been criticized for incomplete evaluation factors, poor clinical applicability, or mismatched tumor types (37, 38). Our study integrates oncological characteristics with laboratory findings to comprehensively assess lymph node status. Additionally, we employ multiple validation methods to ensure the reliability of our results.

Our developed predictive model exhibits promising potential and practicality for clinical treatment decisions. Demonstrating robust discrimination and calibration, the model accurately assesses cervical lymph node status in patients with laryngeal and hypopharyngeal carcinoma, providing valuable insights for clinical decision-making. Moreover, the predictors utilized in the model are based on routine clinical examinations, showcasing their broad applicability and utility in everyday clinical practice. This facilitates personalized treatment approaches, ultimately enhancing treatment outcomes.

However, our study does have limitations. Firstly, it is based on single-center cases and lacks external validation, restricting the model’s applicability to broader populations. Additionally, retrospective designs may introduce confounding factors that could affect result accuracy. To address these limitations, we propose conducting prospective studies across multiple centers and populations to validate the research model, ensuring its generalizability. Furthermore, we suggest exploring the integration of tumor biomarkers and genomic data into the predictive model. By incorporating these biomarkers and genetic information, we can enhance the accuracy and precision of assessing lymph node status in patients. Additionally, investigating the potential synergy between traditional clinical variables and novel biomarkers warrants further exploration. These approaches hold promise for developing comprehensive and precise predictive models, thereby improving clinical outcomes for patients.

## Conclusion

We have developed and validated a diagnostic prediction model that can predict lymph node status using non-invasive methods before treatment, addressing the clinical challenge of detecting occult neck lymph node metastasis. This model holds promise for clinical application, providing valuable insights for patient management.

## Data availability statement

The original contributions presented in the study are included in the article/Supplementary Material. Further inquiries can be directed to the corresponding authors.

## Ethics statement

The studies involving humans were approved by ethics committee of Army Medical Center of PLA. The studies were conducted in accordance with the local legislation and institutional requirements. The ethics committee/institutional review board waived the requirement of written informed consent for participation from the participants or the participants’ legal guardians/next of kin because retrospective study approved by the ethics committee.

## Author contributions

XDW: Conceptualization, Data curation, Formal analysis, Funding acquisition, Investigation, Methodology, Project administration, Resources, Software, Supervision, Validation, Visualization, Writing – original draft, Writing – review & editing. YX: Conceptualization, Data curation, Formal analysis, Funding acquisition, Investigation, Methodology, Project administration, Resources, Software, Supervision, Validation, Visualization, Writing – original draft, Writing – review & editing. WZ: Conceptualization, Data curation, Formal analysis, Investigation, Methodology, Project administration, Software, Supervision, Validation, Writing – original draft. XPW: Conceptualization, Data curation, Formal analysis, Investigation, Methodology, Project administration, Software, Supervision, Validation, Writing – original draft. JC: Conceptualization, Data curation, Formal analysis, Investigation, Methodology, Project administration, Resources, Software, Supervision, Validation, Visualization, Writing – original draft, Writing – review & editing. GL: Conceptualization, Data curation, Formal analysis, Investigation, Methodology, Project administration, Resources, Software, Supervision, Validation, Visualization, Writing – original draft, Writing – review & editing.
